# Fractalkine Depresses Cardiomyocyte Contractility

**DOI:** 10.1371/journal.pone.0069832

**Published:** 2013-07-30

**Authors:** David Taube, Jiang Xu, Xiao-Ping Yang, Albertas Undrovinas, Edward Peterson, Pamela Harding

**Affiliations:** 1 Hypertension and Vascular Research Division, Henry Ford Hospital, Detroit, Michigan, United States of America; 2 Cardiovascular Research Division, Henry Ford Hospital, Detroit, Michigan, United States of America; 3 Department of Internal Medicine and Department of Public Health Sciences, Henry Ford Hospital, Detroit, Michigan, United States of America; Institute of Clinical Medicine, National Cheng Kung University, Taiwan

## Abstract

**Background:**

Our laboratory reported that male mice with cardiomyocyte-selective knockout of the prostaglandin E_2_ EP4 receptor sub-type (EP4 KO) exhibit reduced cardiac function. Gene array on left ventricles (LV) showed increased fractalkine, a chemokine implicated in heart failure. We therefore hypothesized that fractalkine is regulated by PGE_2_ and contributes to depressed contractility via alterations in intracellular calcium.

**Methods:**

Fractalkine was measured in LV of 28–32 week old male EP4 KO and wild type controls (WT) by ELISA and the effect of PGE_2_ on fractalkine secretion was measured in cultured neonatal cardiomyocytes and fibroblasts. The effect of fractalkine on contractility and intracellular calcium was determined in Fura-2 AM-loaded, electrical field-paced cardiomyocytes. Cardiomyocytes (AVM) from male C57Bl/6 mice were treated with fractalkine and responses measured under basal conditions and after isoproterenol (Iso) stimulation.

**Results:**

LV fractalkine was increased in EP4 KO mice but surprisingly, PGE_2_ regulated fractalkine secretion only in fibroblasts. Fractalkine treatment of AVM decreased both the speed of contraction and relaxation under basal conditions and after Iso stimulation. Despite reducing contractility after Iso stimulation, fractalkine increased the Ca^2+^ transient amplitude but decreased phosphorylation of cardiac troponin I, suggesting direct effects on the contractile machinery.

**Conclusions:**

Fractalkine depresses myocyte contractility by mechanisms downstream of intracellular calcium.

## Introduction

Inflammation is a crucial component in the process of heart failure. However, the precise pathways linking the two are unknown. Chemokines are a super-family of small (8–10 kDa), inducible, secreted pro-inflammatory cytokines that act primarily as chemoattractants and activators of specific leukocytes [1], [2]. Fractalkine is the only chemokine in the class of Cx3C chemokines and is unique in that it can be either membrane-tethered or released as a secreted protein. The membrane bound form is believed to mediate cell-cell interactions and may act as a reservoir for the secreted form. Soluble fractalkine is secreted mainly by endothelial cells and is a chemoattractant for monocytes/macrophages, natural killer cells, T lymphocytes and vascular smooth muscle cells, all of which express Cx3CR1, its receptor [3]. These chemokines and their receptors are induced upon T cell activation and by cytokines such as interleukin-1 beta (IL-1β) and gamma interferon (IFNγ). The contribution of resident cardiac cells to the elaboration of these chemokines and their role in pathophysiology is not well-studied but recent evidence shows that both cardiomyocytes and fibroblasts are capable of secreting fractalkine and express its receptor [4]. In a rat model of experimental myocarditis, fractalkine mRNA was detected both in cardiomyocytes and in non-myocyte cells whereas its receptor was found on inflammatory cells suggesting that the increase on resident cardiac cells is responsible for attraction of inflammatory CD4+ T cells and CD11b+ monocytes and macrophages [5]. Furthermore, up-regulation of fractalkine and its receptor on cardiomyocytes was strongly associated with the degree of heart failure in both human and animal models [6] and Richter et al identified fractalkine as an independent predictor of mortality in patients with advanced heart failure [7]. Together, these papers underscore a role for chemokines and other immune modulators in the pathogenesis of heart failure. A role for fractalkine was shown in cardiac allograft rejection whereby mice lacking the receptor exhibited a longer graft survival time accompanied by a reduction in macrophages, natural killer cells and other leukocytes [8]. Fractalkine was also shown to play a major role in macrophage infiltration through the atrial endocardium of rats subject to LPS-induced inflammation [9]. Furthermore, disruption of the fractalkine receptor reduced the number of macrophages and their products (e.g.TGFα, VEGF) along with collagen deposition in a mouse model of wound repair [10]. Interestingly, TNFα induces fractalkine expression in monocytes and TNFα-stimulated adhesion to endothelial cells was partially blocked by an anti-fractalkine antibody [11]. Administration of cDNA encoding chemokine receptors (CCR2 and CxCR3) prevented dilated cardiomyopathy and death in a model of myocarditis, presumably acting as a decoy receptor [12] and targeted deletion of CCR2 reduced ventricular remodeling after experimental MI [13]. However, many of these previous studies did not elucidate whether resident cardiac cells contribute to fractalkine production or whether fractalkine has a role in cardiac function beyond its role as a chemoattractant.

Our laboratory has previously reported that fractalkine mRNA is upregulated in hearts of 28–32 week old male mice with cardiomyocyte-selective knockout of the prostaglandin E_2_ EP4 receptor sub-type (EP4 KO) [14], suggesting for the first time that fractalkine may be regulated by PGE_2_. These EP4 KO mice exhibit reduced cardiac function and a phenotype resembling that of dilated cardiomyopathy [14]. However, our previous study did not address whether this particular chemokine has direct effects on cardiac function.

Reduced contractility of the cardiac myocyte is a hallmark feature of heart failure. Although it is well appreciated that changes in either the size or duration of the calcium transient can greatly affect contractility, it is also understood that alterations in myofilament proteins or their properties can impact cardiac contraction. Cardiac troponin I is the inhibitory sub-unit of the troponin complex of the thin filament and, as such, is an important regulator of contraction. At diastolic levels of intracellular calcium, troponin I inhibits actin-myosin interactions whereas the binding of calcium to troponin C during systole induces a conformational change that relieves the inhibitory effect of troponin I, promotes actin-myosin cross bridge formation and contraction ensues. As calcium levels fall during diastole, calcium dissociates from troponin C and the inhibitory effect of troponin I is restored. Phosphorylation on different serine and threonine residues has been shown to regulate myofilament properties. PKA dependent phosphorylation of troponin I decreases myofilament calcium sensitivity which increases the rate of dissociation of Ca^2+^ from troponin C and accelerates relaxation. Currently few studies have addressed whether chemokines can modulate contractility and their mechanism of action. Thus, the present study examines whether fractalkine can directly affect myocyte contractility under basal conditions or after beta adrenergic stimulation. Additionally, this study examines whether fractalkine is regulated by PGE_2_ in cardiomyocytes and fibroblasts. Our novel findings demonstrate that fractalkine depresses myocyte contractility and uncovers a new mechanism of action for this chemokine.

## Materials and Methods

### Materials

Mouse recombinant fractalkine was from R & D Systems (Minneapolis, MN). The EP4 agonist, AE1-329 was a generous gift from ONO pharmaceuticals (Osaka, Japan) and sulprostone was from Cayman Chemical (Ann Arbor, MI). Isoproterenol was from Sigma and Fura-2AM was from Molecular Probes (Eugene, OR). Collagenase type II was from Worthington (Lakewood, NJ) and protease type XIV from Streptomyces griseus was from Sigma (St Louis, MO).

### Animal Use

The wild type and EP4 KO mice used in this study were bred and genotyped at Henry Ford Hospital and have been previously described [14]. C57Bl/6 mice used for the contractility studies were from Jackson labs. The isolation of neonatal rat ventricular cardiomyocytes (NVM) and fibroblasts (NVF) along with adult mouse ventricular cardiomyocytes (AVM) was previously described by us [14], [15]. All studies involving the use of animals were approved by the institutional review committee at Henry Ford Hospital, in accordance with federal guidelines.

### Echocardiography of Mice

Echocardiography was performed on conscious male WT and EP4 KO animals at 28 weeks of age. The cardiac function of all mice was assessed by echocardiography using an Acuson 256 system (Mountain View, CA) with a 15-MHz linear transducer, as reported previously [14]. Mice were conscious during the procedure. Diastolic measurements were made at the maximum left ventricle cavity dimension, whereas systolic parameters were measured during maximum anterior motion of the posterior wall. All echocardiography was performed by the same investigator who was blinded to the genotype.

### Fractalkine Real Time RT-PCR and ELISA

Fractalkine mRNA was measured by quantitative real-time RT-PCR using a SYBR green method. Predesigned mouse-specific primers from SA Biosciences (Frederick, MD) were used for all PCR reactions. One microgram of DNase-treated total RNA sample was reverse transcribed using random primers and Omniscript reverse transcriptase (Qiagen, Valencia, CA). Two microliters of the reverse transcription reaction were then amplified in a Roche version 2.0 LightCycler PCR instrument (Indianapolis, IN) using SYBR green dye (SA Biosciences) and specific primers according to the SA Biosciences protocol. At the end of PCR cycling, melting curve analyses were performed and representative PCR products were run on agarose gels and visualized by ethidium bromide staining. RT-PCR of GAPDH was used for normalization of all data. A relative quantitation method (ΔΔC_t_) [16] was used to evaluate expression of each gene in KO heart relative to WT.

Expression of the fractalkine receptor (CX3CR1) was also measured in NVM, NVF and AVM by semi quantitative RT-PCR. Reverse transcription was performed as described above and PCR was carried out for a total of 40 cycles with annealing at 58°C using the following primer sequences: Rat forward primer 5′ cccagctgctcaggac 3′, mouse forward primer 5′ cttcccatctgctcaggac and rat/mouse reverse primer 5′ ccaccagaccgaacgt 3′.Fractalkine protein was measured in left ventricle homogenates by an ELISA from RayBio (Norcross, GA) with a standard curve ranging from 2.74–2000 pg/ml. Briefly, male 28–29 week old WT and EP4 KO mice were anesthetized with Nembutal and their hearts removed. The hearts were briefly washed in ice-cold PBS and the right ventricle was dissected free of the septum. The left ventricle free wall plus septum was then homogenized in 200 µl of assay buffer from the kit with the addition of protease inhibitors (Complete Mini-EDTA free protease inhibitor cocktail tablets, Roche). Homogenates were then centrifuged at 10,000 rpm for 10 min at 4°C, the supernatant removed and stored at −80°C prior to ELISA. All values were normalized to protein content.

For the cell culture studies to investigate the effect of PGE_2_ and the EP4 agonist on fractalkine secretion, we used primary cultures of NVM plated at a density of 1×10^6^ cells/well in 6 well plates or primary cultures of NVF at passage 2 (P2) that were approximately 80% confluent. Cells were serum starved for 24 hrs and then changed to fresh serum-minus media. For the experiments on NVM using hydrogen peroxide, cells were treated with either PGE_2_ or the EP4 agonist for 30 min prior to stimulation for a further 90 min. Experiments to determine the effect of PGE_2_ and the EP4 agonist on fractalkine secretion by NVF cultures were performed after 24 hrs of treatment. At the end of the experimental period, media was removed and snap-frozen in liquid N2, then stored at −80°C before assaying for fractalkine.

### Isolation of Adult Cardiomyocytes for Contractility Studies

Isolation of cardiomyocytes from 16–20 week- old C-57 adult male mouse hearts (n = 9) was performed using modifications of the method described by O’Connell et al [17] and has previously been described by us [14]. 10 mmol/l 2,3-butanedione monoxime was omitted as it is a known inhibitor of contractility.

### Determination of Cardiomyocyte Contractility and Intracellular Calcium

Freshly isolated AVM prepared in Tyrode’s solution were loaded with1 µmol/L Fura-2 AM (Molecular Probes, Eugene, OR) for 5 min at room temperature, washed and rested for 15 minutes. After cells were loaded and rested, cardiomyocytes were divided into aliquots and treated with vehicle or 5 ng/ml fractalkine for 10 minutes and washed. An aliquot of cells was added to the chamber and cells were allowed to attach for 2 min, then superfused with Tyrode’s solution at 37°C and electrically stimulated at 3 Hz using a biphasic pulse. The response to isoproterenol was assessed by changing the perfusion buffer to contain 0.1 µmol/L isoproterenol. For the isoproterenol experiments, data for analysis was taken from the point before agonist application and 200 secs after, as described in Pyo et al [18]. Contraction amplitude and intracellular calcium transients were recorded online using a dual excitation spectrofluorometer and video edge detection system (IonOptix) and a minimum of 50 transients were analyzed for each cell. As indicators of contractility; peak shortening, and the speed of contraction and relaxation were measured. As an index of intracellular calcium, the calcium transient amplitude as a change in the F340/F380 ratio and the decay time constant, tau [19], [20] was evaluated. In order to ensure that the response to fractalkine was not due to reduced cell viability, in some experiments the cells treated with fractalkine were assessed first and then the cells treated with vehicle were assessed.

### Effect of Fractalkine on Phosphorylation of Troponin I

To determine whether fractalkine depressed contractility of AVM by affecting phosphorylation of troponin I, AVM from 21 week old male C57BL/6 mice were treated with either 5 ng/ml fractalkine or its vehicle for 10 min at room temperature and were then washed and challenged with 0.1 µmol/L Iso for a further 10 min at 37°C. Samples were immediately placed on ice, centrifuged at 5000g for 5 min, the supernatant removed and the pellet resuspended in 200 µl of 1X lysis buffer containing protease and phosphatase inhibitors. The samples were then vortexed and centrifuged at 10,000g for 5 min to remove cellular debris, and the supernatant was removed and stored at −80°C prior to Western blot analysis. For Western blot analysis, 50 µg protein lysates were resolved on a 14% gel under reducing conditions and after electrophoresis, they were transferred overnight to a PVDF membrane. Membranes were blocked for 1 hr in 5% milk (v/v in TBS-Tween) and incubated overnight (4°C) with a polyclonal antibody against phosphorylated cardiac troponin I at Ser ^23/24^(Cell Signaling) at a 1∶1000 dilution in 5% milk. After washing with TBS-tween, membranes were incubated with a HRP-conjugated goat anti-rabbit secondary antibody for 1 hr at room temperature at a dilution of 1∶2000. After further washing they were developed using a Super Signal West Pico chemiluminescent substrate (Pierce, Rockford, IL). An antibody against total cardiac troponin I (Cell Signaling) that recognizes both phosphorylated and non-phosphorylated forms was used for normalization and was used at a dilution of 1∶1000 in 5% milk.

### Statistical Analysis

All statistics were performed by a statistician in the Department of Public Health Sciences of Henry Ford Hospital. For the contractility data, statistics are reported as means +/− SEM with ‘n’ representing the number of cells. For all other data, ‘n’ represents the number of experiments. Groups were compared with Student’s t-test except where normality was not present and a two-sample Wilcoxon test was used. Association between variables was assessed using standard Pearson correlation coefficients. A p-value <0.05 was considered as evidence of a statistically significant difference for experimental data.

## Results

### Left Ventricle Fractalkine Protein and Cardiac Performance

**Figure 1:**
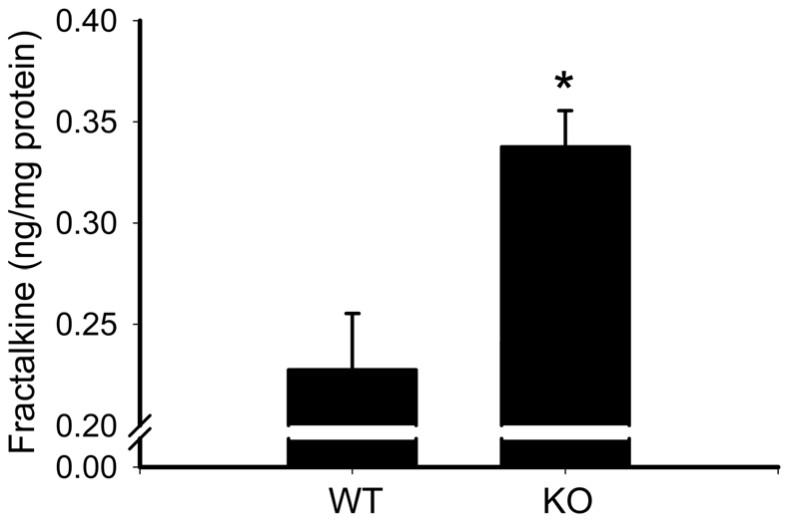
Left ventricle (LV) fractalkine concentration in 28 week old male EP4 KO mice and WT controls. Values are corrected to ng fractalkine per mg LV weight. Fractalkine was determined by ELISA. *p<0.05.

To determine whether increased fractalkine content of the left ventricle was associated with reduced cardiac function, we performed echocardiography and measured fractalkine in the left ventricle of WT and those EP4 KO mice that had a low ejection fraction. As shown in Table 1, these EP4 KO mice show left ventricle chamber dilatation coupled with thinning of the left ventricle wall, a reduced shortening fraction and a reduced ejection fraction; consistent with the phenotype of dilated cardio-myopathy that we have previously reported. Figure 1 shows that LV fractalkine is significantly increased in these EP4 KO mice compared to WT littermates (0.34±0.02 ng/mg LV protein vs 0.23±0.02 ng/mg LV protein, p<0.05), suggesting a correlation between the two parameters.

**Table 1:** Echocardiography of Male EP4 KO Mice and Wild Type Littermates.

Parameter	WT	EP4 KO
BW (g)	37.0±1.10	33.5±0.96
SF (%)	54.4±1.28	38.6±4.88*
EF (%)	85.6±0.52	49.5±7.05**
LVDs (mm)	1.17±0.04	2.29±0.28*
LVDd (mm)	2.57±0.07	3.70±0.23**
HR (bpm)	676±5.0	684±15.1
PWTd (mm)	0.87±0.01	0.82±0.01*
IVSTd (mm)Mass (mm^2^/g)	0.91±0.021.93±0.08	0.82±0.02*3.24±0.29*

Table 1 gives echocardiography data for male EP4 KO mice and their wild-type littermates (WT) that were used to determine fractalkine content of the left ventricle by ELISA. Values are means ± SE.

* p<0.05,

** p≤0.01 versus WT. Statistical analysis was performed using t-test. Abbreviations: SF, shortening fraction; EF, ejection fraction; LVDs, left ventricular dimension at systole; LVDd, left ventricular dimension at diastole; HR, heart rate; PWTd, posterior wall thickness at diastole; IVSTd, intraventricular septum thickness at diastole.

### Effect of PGE_2_ on Fractalkine from Cardiomyocytes (NVM and AVM) and Fibroblasts

To determine whether fractalkine secretion is regulated by PGE_2_ and via what specific EP receptor, we treated primary cultures of NVM with either PGE_2_ (10^−6^, 10^−8^ mol/L), the EP1/EP3 agonist sulprostone (10^−6^ mol/L) or the EP4 agonist ONO AE1-329 (10^−6^, 10^−8^ mol/L) for 2 hrs and measured fractalkine secretion in the media with an ELISA kit for rat fractalkine (RayBio, Norcross, GA). As oxidative stress is known to increase fractalkine in other cell types, we also examined the effect of 1 mM hydrogen peroxide on fractalkine secretion from NVM and determined whether this was affected by pre-treatment with either the EP4 agonist or PGE_2_. Our data indicate that neither PGE_2_ nor the EP4 agonist significantly affect fractalkine secretion from NVM under basal conditions. Treatment of NVM with hydrogen peroxide significantly increased fractalkine secretion from 243.9±34.2 pg/ml to 470.9±65.0 pg/ml, p<0.01 but this was unaltered by pre-treatment with either PGE_2_ (460.4±64.0 pg/ml) or the EP4 agonist (463.1±73.1 pg/ml).

**Figure 2:**
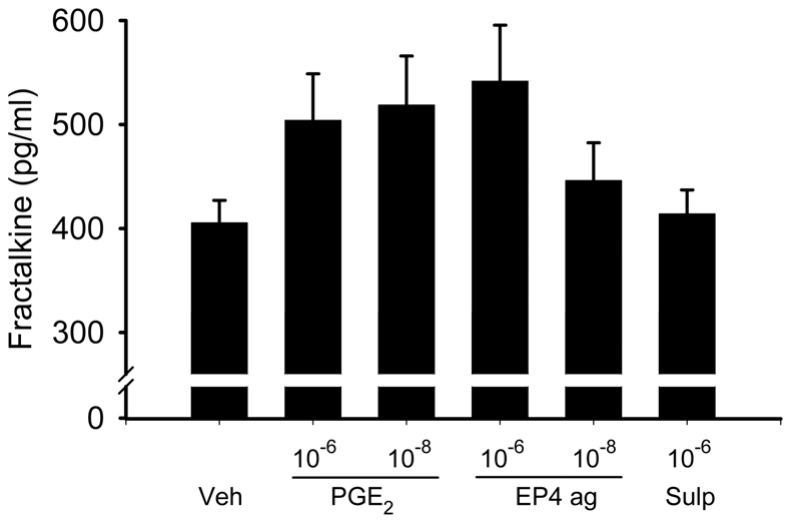
Effect of PGE_2_ (10^−6^ M, 10^−8^ M), the EP4 agonist (EP4 ag,10^−6^ M, 10^−8^ M), and sulprostone (sulp,10^−6^ M) on fractalkine secretion from NVF. Cells at passage two were treated with the various compounds for 24 hr and fractalkine secretion into the media was measured by ELISA. Statistical significance: *p<0.05 compared to vehicle control. N = 6.

To determine whether PGE_2_ regulated fractalkine secretion from fibroblasts, we performed experiments on NVF at passage 2. Figure 2 shows that treatment of NVF for 24 hr with either 10^−6^ mol/L or 10^−8^ mol/L PGE_2_ increased fractalkine secretion from 405.5±21.5 pg/ml to 504±44.7 pg/ml, p = 0.07 and 518.7±47.2 pg/ml, p<0.05 respectively. Likewise, the EP4 agonist (10^−6^ mol/L) also increased fractalkine secretion to 541.7±53.8 pg/ml, p<0.05 but the EP1/EP3 agonist sulprostone had no effect (414.1±23.0 pg/ml).

**Figure 3:**
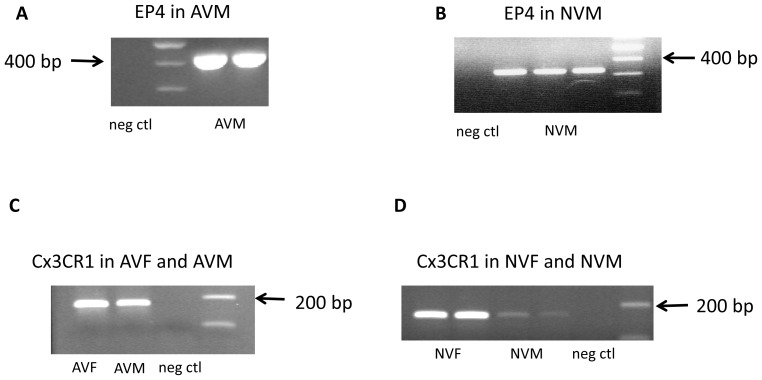
Panels A and B show expression of the EP4 receptor in adult mouse ventricular myocytes (AVM) and neonatal rat ventricular myocytes (NVM), respectively, as determined by RT-PCR. Neg ctl is the negative control lane. Note that the PCR product sizes are different in panel A and panel B because different primer sets were used for the two different species. Panel C shows expression of the fractalkine receptor (Cx3CR1) in adult mouse fibroblasts (AVF) and myocytes (AVM) and panel D shows expression of the fractalkine receptor in cultured neonatal rat ventricular fibroblasts (NVF) at passage two and in primary cultures of neonatal rat ventricular myocytes (NVM). Expression of the fractalkine receptor was also determined by RT-PCR.

Since we were not able to detect an effect of PGE_2_ or the EP4 agonist on fractalkine in NVM, we performed control experiments to determine the presence of the EP4 receptor. As shown in Figure 3, these cultured cardiomyocytes express the EP4 receptor thus the negative result cannot be attributed to a lack of the EP4 receptor. This result also confirms that increased fractalkine in the LV of EP4 KO mice is not from the cardiomyocytes. To confirm that the results obtained in neonatal rat hearts correlated with those of adult mouse hearts, we isolated ventricular cardiomyocytes from C57Bl/6 mice and tested the effect of PGE_2_, the EP4 agonist and sulprostone on fractalkine mRNA using real time RT-PCR. Treatment with these compounds for 24 hrs did not affect fractalkine mRNA levels.

### Effect of Fractalkine on Contractility and Intracellular Calcium

**Figure 4:**
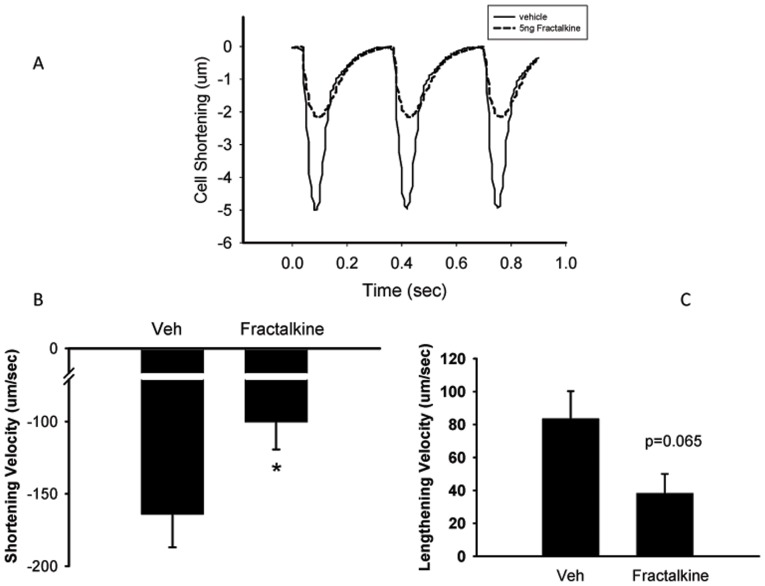
Panel A shows representative transients of cells treated with either vehicle or 5 ng/ml fractalkine for 10 min. y-axis is the magnitude of cell shortening (contraction) in µm. Cells were paced at 3 Hz. Panels B and C show mean data for the effect of 5 ng/ml fractalkine on shortening velocity and lengthening velocity under basal conditions, respectively. Statistical significance: *p<0.05 compared to cells treated with vehicle. N = 22–33 cells from 9 mice.

**Figure 5:**
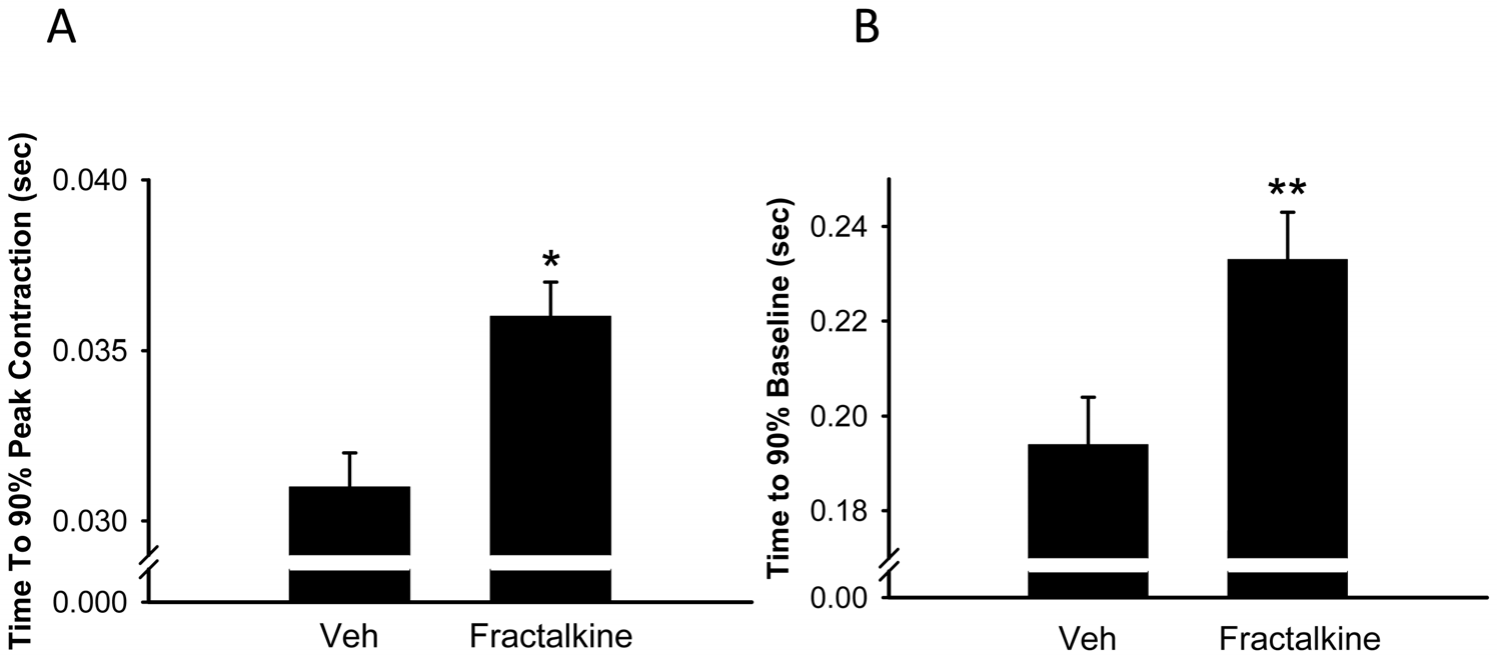
Effect of 5 ng/ml fractalkine on time to 90% peak contraction (panel A) and time to 90% of baseline (panel B) under basal conditions. Statistical significance: *p<0.05, **p<0.01 compared to cells treated with vehicle.

**Figure 6:**
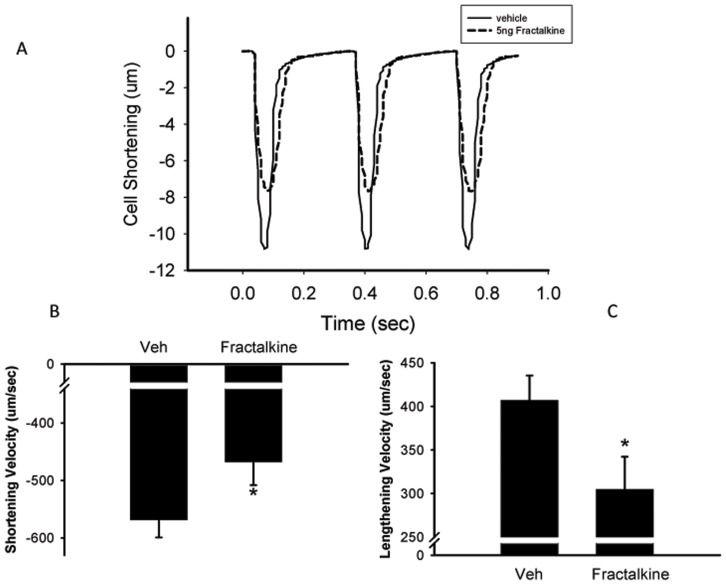
Panel A shows representative transients from vehicle and fractalkine-treated cells under isoproterenol-stimulated conditions. y-axis is the magnitude of cell shortening (contraction) in µm. Cells were paced at 3 Hz. Panels B and C show mean data for the effect of 5 ng/ml fractalkine on shortening velocity and lengthening velocity, respectively under isoproterenol-stimulated conditions. Statistical significance: *p<0.05 compared to cells treated with vehicle. N = 22–33 cells from 9 mice.

**Figure 7:**
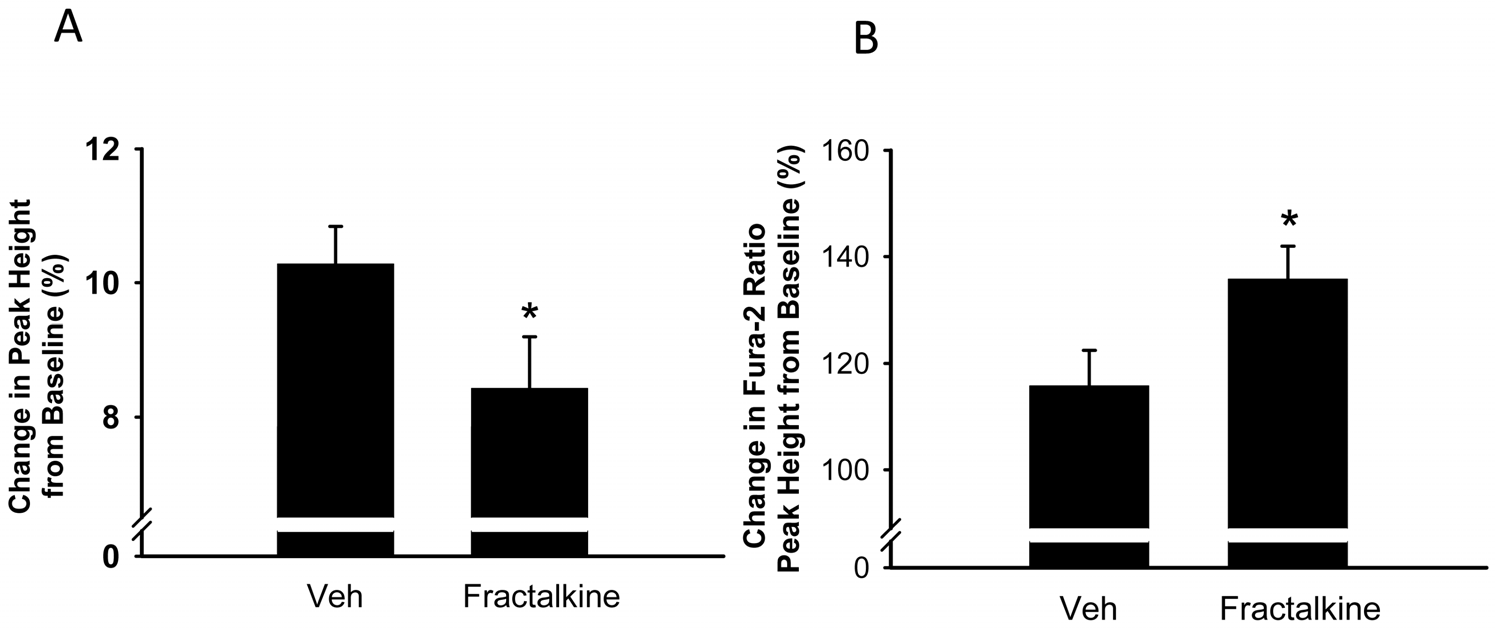
Effect of 5 ng/ml fractalkine on change in peak height from baseline (%) under isoproterenol-stimulated conditions (panel A) and change in intracellular calcium as indicated by a change in the Fura-2 ratio (panel B). Statistical significance: *p<0.05 compared to cells treated with vehicle.

Figures 4B and 4C show that under basal conditions, treatment of AVM for 10 min with 5 ng/ml fractalkine decreased both the speed of contraction and relaxation (from −164.1±22.8 to −100.4±18.9 µm/sec, p<0.05; and from 83.3±16.8 to 38.2±11.7 µm/sec respectively, p = 0.065). Figure 4A shows representative transients from one cell treated with vehicle and one cell treated with fractalkine. When the data was averaged from 50–100 transients per cell from a total of 22–33 cells (9 mice), mean peak contraction was 3.15±0.5 in vehicle-treated cells and was reduced to 1.93±0.4 in fractalkine-treated cells, although this failed to achieve statistical significance, p = 0.13.Treatment with fractalkine also increased the time taken for 90% of the peak contraction and relaxation (Fig. 5). Experiments to examine the effect of fractalkine on contractile function were also performed under beta adrenergic stimulation with isoproterenol, an agent that is known to increase the speed and force of contraction. A representative transient under isoproterenol-stimulated conditions is seen in Figure 6A. A comparison of Figure 6A with that of Figure 4A, clearly indicates that isoproterenol increases the contraction of adult mouse myocytes. After stimulation with isoproterenol, fractalkine also decreased both the speed of contraction and relaxation, from −568.8±30.6 to −467.5±40.5 µm/sec and from 407.0±28.4 to 304.7±37.5 µm/sec respectively, p<0.05 (Figs. 6B and 6C). In addition, fractalkine decreased the percent change in contraction after isoproterenol stimulation from 10.3±0.5 to 8.4±0.8, p = 0.05 (Fig. 7A). Surprisingly, fractalkine increased the percent change in the Fura-2 ratio after isoproterenol stimulation from 115.8±6.6 to 135.8±6.2, p<0.05 despite reducing contractility (Fig. 7B). The decline in the calcium transient, Tau, was not significantly different between vehicle-treated and fractalkine-treated cells after Iso stimulation (sin exp tau was 0.050±0.001 for vehicle treated cells, 0.053±0.001 for fractalkine treated cells).

### Effect of Fractalkine on Phosphorylation of Troponin I

**Figure 8:**
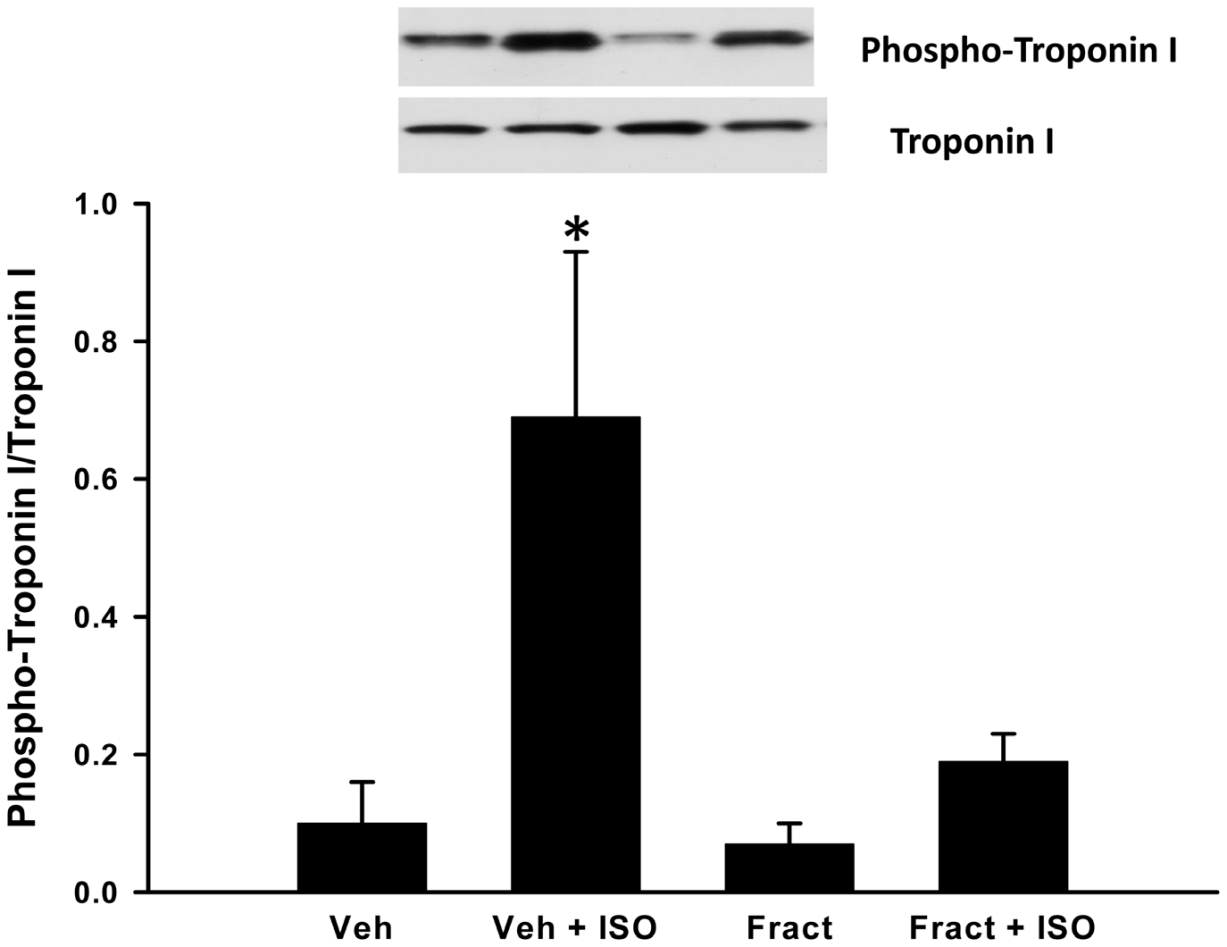
Effect of 5 ng/ml fractalkine on phosphorylation of cardiac troponin I. Panel A shows a representative Western blot of phosphorylated cardiac troponin I (upper panel) and total troponin I after treatment of AVM with vehicle (veh), isoproterenol (Iso), fractalkine (fract) or isoproterenol+fractalkine. N = 4 separate experiments. Statistical significance: *p<0.05 compared to vehicle treatment, +p<0.05 compared to isoproterenol treatment.

To determine whether fractalkine affects the contractile machinery of cardiomyocytes, we measured the phosphorylation of cardiac troponin I under basal conditions and after isopoterenol stimulation as described in the methods section. As shown in Figure 8, treatment with isoproterenol elicited the expected increase in phosphorylation of cardiac troponin I and this effect was antagonized by pre-treatment with fractalkine. Phospho troponin I, corrected to total troponin I, was 0.10±0.06 under vehicle-treated conditions, 0.69±0.24 after stimulation with isoproterenol and was 0.19±0.04 in isoproterenol stimulated cells pre-treated with fractalkine. However, fractalkine treatment did not significantly affect phosphorylation of cardiac troponin I under basal conditions (0.10±0.06 vs 0.07±0.03 units).

### Expression of the Fractalkine Receptor (Cx3CR1)

To confirm the presence of the fractalkine receptor on cardiomyocytes and fibroblasts, we performed RT-PCR as described in the methods section. As shown in Figure 3C and 3D, we were able to detect robust expression of the receptor in adult mouse ventricular fibroblasts and cardiomyocytes. We were also able to detect the receptor in cultured neonatal rat ventricular fibroblasts and cardiomyocytes, albeit with much less expression in the latter.

## Discussion

Although the chemotactic effects of fractalkine are well-known, whether or not it regulates function of the cardiomyocyte is less clear and understudied. The results of our study demonstrate, for the first time, that fractalkine reduces single myocyte contractility after beta adrenergic stimulation and is regulated by PGE_2_ in fibroblasts but not cardiomyocytes. Importantly, we show here for the first time that fractalkine decreases phosphorylation of cardiac troponin I in AVM after isoproterenol stimulation; thus providing a potential mechanism for the effects of fractalkine on isoproterenol-induced contractility.

To determine whether PGE_2_ down-regulation of fractalkine accounts for its increase in the left ventricle of EP4 KO mice, we examined the effect of PGE_2_, an EP1/EP3 agonist and an EP4 agonist on fractalkine secretion by NVM and NVF. Our data do not support a role for PGE_2_ in decreasing fractalkine secretion in cardiomyocytes; indeed an opposite trend was noted. However, both PGE_2_ and the EP4 agonist stimulated secretion in fibroblasts suggesting either that the source of increased fractalkine in the left ventricle of cardiomyocyte-specific EP4 KO mice is non-myocyte cells i.e fibroblasts/endothelial cells/infiltrating cells or that a different factor is regulating fractalkine secretion from cardiomyocytes in these failing hearts. Our present data does not allow for this differentiation. We also observed that fractalkine secretion is stimulated by the addition of hydrogen peroxide to cardiomyocytes; suggesting that oxidative stress may lead to production of this chemokine. Whereas fractalkine was previously shown to increase reactive oxygen species in other cell types [21], our data indicate a potential positive feedback loop whereby fractalkine secretion is also increased by oxidative stress. Whether the EP4 KO mice have increased oxidative stress within the heart is unknown but likely since an interstitial inflammatory infiltrate is present in the heart [14] and we previously reported that mice deficient in microsomal prostaglandin E synthase-1 have increased NOX2 in the left ventricle during Ang II-dependent hypertension [22]. Our finding that fractalkine is increased in the left ventricles of EP4 KO mice with impaired cardiac function is in agreement with the findings of Xuan et al [4] who described that fractalkine was increased in heart failure induced by trans-aortic constriction or MI and that the outcome of these disease processes could be improved by a neutralizing antibody to fractalkine. Whether or not a similar approach would be beneficial in our EP4 KO mice remains to be determined.

Protein kinase A (PKA)-dependent phosphorylation of cardiac troponin I on Serine 23 and 24 residues was postulated to play a role in enhancing the rate of contraction and relaxation after beta adrenergic stimulation [23] and decreased phosphorylation of cardiac troponin I was observed in patients with heart failure [24], [25]. Our finding that fractalkine, at a concentration found in heart failure patients, can dramatically impair contractility is significant. Indeed, the reduced rate of contraction and relaxation observed after treatment with this chemokine would indicate that it can lead to systolic and diastolic dysfunction. Although it was surprising that fractalkine could reduce contractility after isoproterenol stimulation without decreasing intracellular calcium, other investigators have reported such examples [26] and our finding that fractalkine decreased phosphorylation of cardiac troponin I after β-adrenergic stimulation is novel and suggests direct effects of this chemokine on the contractile apparatus. The addition of fractalkine to neonatal cardiomyocytes was reported to increase hypertrophic markers and the phosphatases PP2A and PP1, known regulators of cardiac function [6] but whether fractalkine reduces isoproterenol-stimulated troponin I phosphorylation by increasing expression and/or activity of these phosphatases remains to be elucidated. Moreover, our results show that fractalkine does not reduce phosphorylation of cardiac troponin I under basal conditions, perhaps suggesting a different mechanism for the depressor effects of fractalkine under basal conditions. Whether effects on other contractile proteins are responsible for this remain under investigation.

**Figure 9:**
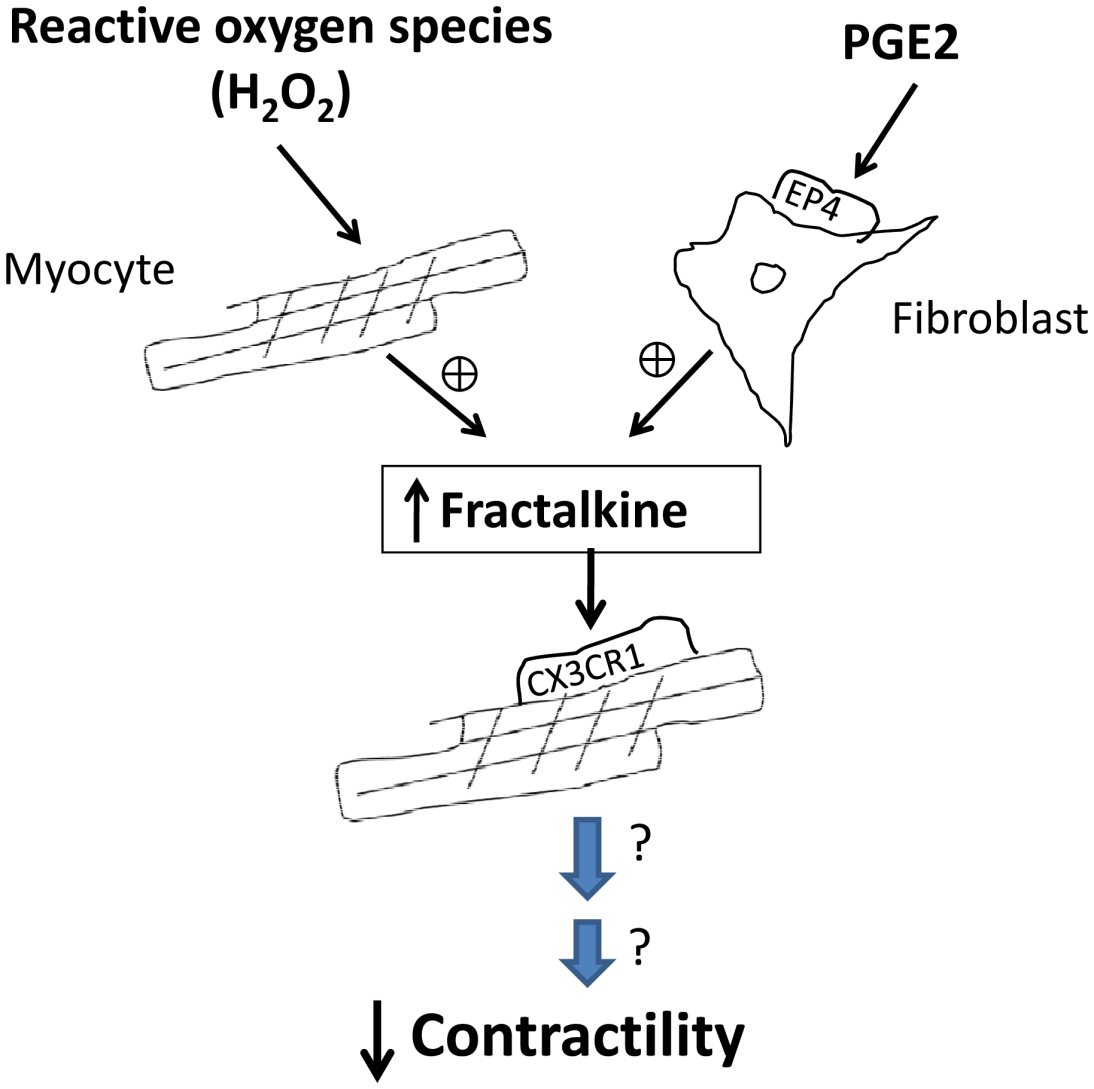
Scheme showing the proposed relationship between PGE_2_, fractalkine and decreased contractility. We propose that PGE_2_ acting on its EP4 receptor on cardiac fibroblasts increases fractalkine secretion. Fractalkine binds to its receptor (Cx3CR1) on cardiomyocytes thereby initiating a chain of events leading to decreased contractility. Also shown is the stimulation of fractalkine secretion from cardiomyocytes by hydrogen peroxide (reactive oxygen species, ROS), a mechanism that appears to be independent of PGE_2_.

The concept that chemokines can directly affect myocyte contractility is relatively new. To our knowledge there are few published papers describing that chemokines decrease contractility of isolated cardiomyocytes. Pyo et al [18] reported that CxCL12 (also known as SDF-1α) decreased contractility via its receptor CxCR4 and more recently, an elegant paper by LaRocca et al [27] reported that activation of CxCR4 by CxCL12 antagonizes beta adrenergic stimulation of PKA by a physical interaction between CxCR4 and the β2-adrenergic receptor that alters G-protein coupled receptor signaling. In a different study, fractalkine was also shown to decrease contractility in response to beta adrenergic stimulation [28]; however, that study used only the number of beats per minute to assess contractility of neonatal cardiomyocytes. Thus, our data showing that fractalkine decreases the speed of contraction and relaxation of adult ventricular cardiomyocytes under both basal conditions and after adrenergic stimulation, measuring cell shortening and intracellular calcium, is novel. Additionally, our study confirms the presence of the fractalkine receptor on cardiomyocytes suggesting that fractalkine released from fibroblasts could have a paracrine effect, binding to its receptor on cardiomyocytes to reduce contractility (Figure 9).

Only recently has the interaction between prostanoids and chemokines been studied and the results are not completely uniform. A lack of EP4 receptors on bone marrow derived cells enhanced inflammation in atherosclerotic lesions [29] and an EP4 agonist was effective in reducing inflammation of the brain induced by LPS [30]. An EP4 receptor associated protein attenuated macrophage activation [31] while over-expression of either EP2 or EP4 reduced MCP-1 (CCL2) expression in LPS-stimulated glomeruli [32]. An EP4 agonist reduced cytokine secretion in animal models of cardiac injury, but neither the mechanism nor the responsible cell type was evaluated. In contrast, a COX-2 inhibitor suppressed autoimmune myocarditis in rats by altering the balance of Th1 and Th2 cytokines [33] whereas an EP4 agonist inhibited acute cardiac allograft rejection with a reduction in pro-inflammatory cytokines and chemokines *in vivo* [34]. Thus, the anti-inflammatory effects of PGE_2_ may be dependent on involvement of multiple EP receptors [35]. Our results showing that PGE_2_ and the EP4 agonist increase fractalkine secretion from cardiac fibroblasts would indicate a pro-inflammatory role for PGE_2_ and EP4 in this setting. In conclusion, our results indicate that fractalkine can directly depress myocyte contractility in addition to its known chemotactic effects. This may have significance in heart failure patients who show elevations in this chemokine.
