# Correction: Fractalkine Depresses Cardiomyocyte Contractility

**DOI:** 10.1371/annotation/8ea7695c-59d1-43c4-809b-fe021e07c799

**Published:** 2013-09-24

**Authors:** David Taube, Jiang Xu, Xiao-Ping Yang, Albertas Undrovinas, Edward Peterson, Pamela Harding

Affiliations numbers 1-3 for all authors were given incorrectly. The correct affiliations are: 1 Hypertension and Vascular Research Division, Department of Internal Medicine, Henry Ford Hospital, Detroit, Michigan, United States of America; 2 Cardiovascular Research Division, Department of Internal Medicine, Henry Ford Hospital, Detroit, Michigan, United States of America; 3 Department of Public Health Sciences, Henry Ford Hospital, Detroit, Michigan, United States of America. 

